# Error management theory and the adaptive significance of transgenerational maternal‐stress effects on offspring phenotype

**DOI:** 10.1002/ece3.4074

**Published:** 2018-06-02

**Authors:** Michael J. Sheriff, Ben Dantzer, Oliver P. Love, John L. Orrock

**Affiliations:** ^1^ Department of Ecosystem Science and Management Huck Institute of the Life Sciences Pennsylvania State University University Park Pennsylvania; ^2^ Departments of Psychology, Ecology and Evolutionary Biology University of Michigan Ann Arbor Michigan; ^3^ Department of Biological Sciences University of Windsor Windsor ON Canada; ^4^ Department of Integrative Biology University of Wisconsin Madison Wisconsin

**Keywords:** developmental plasticity, maternal effects, maternal programming, maternal stress effects, predictive adaptive responses, signal detection theory

## Abstract

It is well established that circulating maternal stress hormones (glucocorticoids, GCs) can alter offspring phenotype. There is also a growing body of empirical work, within ecology and evolution, indicating that maternal GCs link the environment experienced by the mother during gestation with changes in offspring phenotype. These changes are considered to be adaptive if the maternal environment matches the offspring's environment and maladaptive if it does not. While these ideas are conceptually sound, we lack a testable framework that can be used to investigate the fitness costs and benefits of altered offspring phenotypes across relevant future environments. We present error management theory as the foundation for a framework that can be used to assess the adaptive potential of maternal stress hormones on offspring phenotype across relevant postnatal scenarios. To encourage rigorous testing of our framework, we provide field‐testable hypotheses regarding the potential adaptive role of maternal stress across a diverse array of taxa and life histories, as well as suggestions regarding how our framework might provide insight into past, present, and future research. This perspective provides an informed lens through which to design and interpret experiments on the effects of maternal stress, provides a framework for predicting and testing variation in maternal stress across and within taxa, and also highlights how rapid environmental change that induces maternal stress may lead to evolutionary traps.

## 1 INTRODUCTION

Changes in the parental phenotype can act as a signal to offspring about the future environment that they will encounter and these parental cues can induce adaptive plasticity in offspring characteristics (adaptive transgenerational phenotypic plasticity or adaptive parental effects). Recently, this phenomenon has been increasingly studied in vertebrates in the context of maternal‐stress effects, largely because the maternal phenotype or cue that may induce plasticity in offspring traits (maternal stress hormone levels) is both measurable and amenable to experimental manipulations. In vertebrates, exposure to maternally derived stress hormones (glucocorticoids; i.e., “maternal stress”) is increasingly recognized as a significant factor mediating transgenerational phenotypic plasticity in offspring (Barbazanges, Piazza, Moal, & Maccari, 1996; Gluckman, Hanson, & Spencer, 2005; Love, McGowan, & Sheriff, 2013; Meaney, Szyf, & Seckl, 2007). The consequences of maternal stress have long been considered to be maladaptive in biomedical fields because offspring phenotypes that can occur in response to maternal stress (e.g., smaller size, slower growth, lower energetic demand, higher anxiety‐like behavior) are assumed to confer reduced fitness (Sheriff & Love, 2013). However, researchers have recently proposed that maternal stress can play adaptive roles across a wide variety of animal taxa if stress‐induced phenotypes better prepare offspring for a stressful postnatal environment in mammals (Bian et al., 2015; Dantzer et al., 2013; Sheriff, 2015; Sheriff, Krebs, & Boonstra, 2010), birds (Chin et al., 2009; Coslovsky & Richner, 2011; Love, Chin, Wynne‐Edwards, & Williams, 2005; Love & Williams, 2008), reptiles (Bestion, Clobert, & Cote, 2015; de Fraipont, Clobert, John‐Adler, & Meylan, 2000; Meylan & Clobert, 2005), and fish (Giesing, Suski, Warner, & Bell, 2011). Despite this recent progress, a unified framework that both explains the selective mechanisms and allows field‐testing of the adaptive role of maternal stress has yet to be proposed.

Recent theoretical models and meta‐analysis have been generated to examine the evolution of parental and maternal effects generally (e.g., Kuijper & Hoyle, 2015; Leimar & McNamara, 2015; Uller, Nakagawa, & English, 2013). Using insights from these theoretical models in addition to those from error management theory (EMT; Haselton & Buss, 2000), we provide a framework for generating field‐testable hypotheses regarding the adaptive potential of maternal stress under different scenarios. By providing a mechanistic basis for examining the adaptive potential of maternal‐stress effects (defined as the influence of maternal stress on offspring phenotype), our framework aims to (1) describe how selection pressures can shape these adaptive responses, (2) provide a basis for testing new hypotheses, and overall, (3) catalyze the study of maternal‐stress effects across a diversity of species, life histories, and environments. A strength of our approach is that it provides a means for examining the general maternal stress–offspring phenotype relationship, regardless of whether this relationship is primarily controlled by mothers, offspring, or both. Further, it allows testing of the adaptive potential of maternal stress from the mother's perspective, the offspring's perspective, or both (i.e., does maternal stress increase maternal or offspring fitness or both). We begin by summarizing critical considerations to be appreciated when examining the maternal stress–offspring phenotype relationship. We then outline how applying EMT to transgenerational maternal‐stress effects generates several novel hypotheses and predictions that inform discussions pertaining to the evolution and variation in strength of this relationship across taxa. We finish by using EMT‐generated hypotheses to predict the consequence of this relationship as animals face novel stressors from anthropogenic sources. Although we focus on the maternal stress–offspring phenotype relationship in vertebrates, as this is the area where we feel current paradigms could use productive assessment, our work also has implications for understanding the adaptive value of maternal effects more broadly; we develop this component of our work in our concluding section.

## 2 EVALUATING THE POTENTIAL ADAPTIVE VALUE OF MATERNAL STRESS IN VERTEBRATES

Although the ecology of maternal stress has been an active area of research, the traditional biomedical view that maternal stress generates negative outcomes for both mothers and offspring (i.e., is maladaptive) often still prevails (Sheriff & Love, 2013). Indeed, stress‐induced offspring phenotypes are commonly perceived to have a lower phenotypic quality (i.e., smaller size, slower growth, altered behavior/physiology), generating assumptions that performance in nature will likewise be impaired, and often leaving potential context‐specific benefits untested and therefore underappreciated. This perspective has recently been challenged by ecological hypotheses (e.g., the Environmental Matching Hypothesis; Love & Williams, 2008) and supporting evidence that stress‐induced phenotypes can improve offspring performance in *stressful* (but not benign) postnatal or adult environments (e.g., Dantzer et al., 2013; reviewed in Sheriff & Love, 2013).

To move this field ahead in a productive manner, we suggest that three critical points must be considered prior to assigning any hypothetical adaptive or maladaptive value to maternal‐stress effects (*sensu* Love et al., 2013; Sheriff & Love, 2013; Uller et al., 2013; Sheriff et al., 2017). First, we must appreciate that the value of any phenotype, whether stress‐induced or not, can only be understood by examining performance or fitness in an ecologically relevant context (and not simply assuming the outcome based on the phenotype alone). Second, we must consider the evolutionary and life‐history context of the organism before experiments can be designed to test phenotype‐performance relationships. For example, if predation risk is the most salient selection pressure in the evolution of a species’ stress response, testing phenotypic performance in a food‐restricted environment is unlikely to yield useful inference regarding the fitness value of stress‐mediated offspring plasticity. Finally, we must appreciate that testing phenotypic performance in a singular postnatal environment, particularly if the relative quality of the postnatal environment does not match that of the prenatal environment, is invalid for determining the adaptive potential of maternal stress. For instance, testing the performance of stress‐induced phenotypes relative to control phenotypes in a stressful postnatal environment (and not simply in a control environment) is an absolute requirement for correct inference regarding the adaptive value of stress‐induced plasticity. Stated another way, the fitness outcomes of phenotypes induced by elevated maternal glucocorticoids need to be examined across more biologically and ecologically appropriate environments.

**Figure 1:**
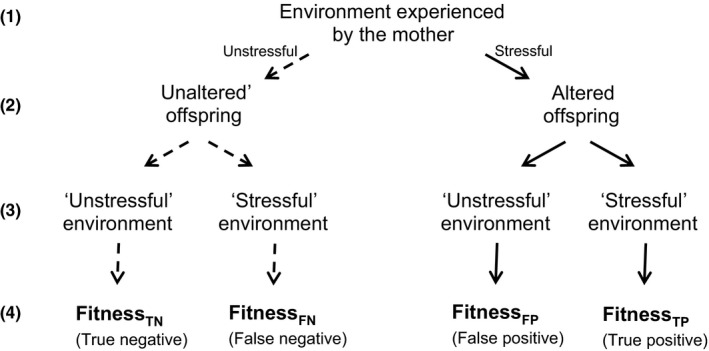
The environment experience by mothers during reproduction can either be unstressful (leading to the dashed arrow pathway) or stressful values (leading to the pathway represented by solid arrows), with the latter occurring when her stress hormone levels are increased beyond some threshold of normal baseline (1). This dichotomy leads to “unaltered” offspring phenotypes or “altered” offspring phenotypes in response to elevated maternal stress (2). These offspring then have the potential to also encounter two different environments; an “unstressful” environment, or, alternatively, a “stressful” environment (3), and their fitness value will depend upon the interaction between their phenotype and the environment they experience (4). We suggest the adaptive potential of maternal stress thus needs to be considered as the *relative* offspring fitness across these scenarios, in a 2 × 2 comparative framework ((*F*_TN_ – *F*_FP_)/(*F*_TP_ – *F*_FN_); Box 1). Additionally, the adaptive potential of maternal stress to maternal (inclusive) fitness can also be evaluated within our framework if the end fitness outcomes (4) are that of the mother (i.e., do mothers survive better and have greater future reproduction if they raise altered offspring in a stressful environment as opposed to attempting to raise unaltered offspring?)

The general under‐appreciation for this latter phenotype‐matching aspect, in particular, is what makes the development of a testable framework to assess the general adaptive potential of maternal stress so valuable. In nature, animals interact with their environments over dynamic spatio‐temporal scales. As such, the quality of the maternal and offspring environment may be temporally or spatially matched, such as may occur in species where there are overlapping generations (temporal matching) or where offspring disperse to areas that are similar to parental environments. Alternatively, past cues may not reliably predict the future (such as in long‐lived animals or those with long‐distance natal dispersal), increasing or decreasing the likelihood that the maternal and offspring environments match (Sheriff & Love, 2013; Sheriff et al., 2017). Thus, to correctly assess the potential adaptive role (if any) of maternal stress, the *relative* offspring phenotype fitness value across biologically relevant environmental scenarios must be examined (Figure 1; Love & Williams, 2008; Uller et al., 2013). Importantly, there are likely very different costs/benefits associated with offspring phenotypic performance depending upon the match or mismatch to future environments (Box 1), and the costs of mismatches, not matches, are expected to play a significant role in the origin and maintenance of transgenerational maternal‐stress effects.

Box 1 **Error management theory and the adaptive role of maternal stress**

1 Selection should favor individuals where maternal stress (e.g., levels of glucocorticoid hormones in vertebrate models) alters offspring phenotype when the benefit of doing so outweighs the costs of not doing so. Because environmental conditions often covary in time and space, current conditions experienced by the mother (i.e., the degree to which the current environment is stressful, represented by the level of maternal stress) may be indicative of conditions that will be experienced by a mother's offspring. If the maternal environment can be used to gauge the future environment, offspring phenotype should be altered at some threshold level (called the decision threshold) where the level of current environmental stressors experienced by the mother has sufficient reliability for predicting likely future environmental stressors for the offspring. The reliability of the current environment to predict the future environment may be indicated by the level of maternal stress hormones. In the figure below, the frequency distributions of the two possible future environments (unstressful or stressful) are plotted against the level of current maternal stress. The level of maternal stress at which offspring phenotype becomes modified determines the relative likelihood of a successful match between offspring phenotype and the type of environment the offspring will experience (i.e., a true positive, TP, or true negative, TN), as well as influences the likelihood of an error, that is, the false positive, FP (unnecessary modification of offspring phenotype), or a false‐negative error (FN, failing to modify offspring phenotype when the future is stressful). Given that the fitness costs of each of these types of error differ (likely such that *F*_TN_ > *F*_TP_ > *F*_FP_ > *F*_FN_; Table 1), and the background probability that the future environment will be stressful (*P*(*s*)) or unstressful (*P*(*ns*)), offspring phenotype should be modified whenever the value of maternal stress is greater than $\frac{P(ns)}{P(s)}\times \frac{\left(F_{\mathrm{TN}}-F_{\mathrm{FP}}\right)}{\left(F_{\mathrm{TP}}-F_{\mathrm{FN}}\right)}$; an example threshold is indicated in the figure below (the vertical line in the middle of the two distributions). The red area to the right of the threshold represents the probabilities of true positives and false positives that would be realized at that particular decision threshold. 
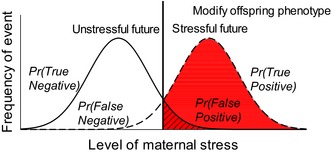

Within the EMT perspective, the costs of TN and TP are often assumed to be identical, as the focus is typically on the evolutionary implications of errors. However, within our framework, and as our matrix shows (Table I), each of the four outcomes is likely to have a different fitness value (indicated by *F*_TN_, *F*_TP_, *F*_FN_, *F*_FP_), because each outcome has a different combination of the two possible error costs. False‐positive errors of producing altered offspring that experience benign environments are expected to be much less costly (in terms of reduced offspring fitness in the benign environment) than false‐negative errors of producing unaltered offspring that experience very stressful environments. However, quantitative assessments of those predictions are rare as few studies perform full factorial experiments in wild animals and assessment of the fitness consequences of false‐positive errors is rare.

In our framework, fitness values can be measured as offspring performance or fitness (e.g., survival), and thus, the relative fitness value of that phenotype can be measured within a given environment. Importantly, our framework can also be expanded to measure relative maternal performance or fitness (e.g., Love, Wynne‐Edwards, Bond, & Williams, 2008) to better understand the adaptive potential of maternal stress for a mother's fitness. This may also allow a comparison of the relative fitness values to the mother and offspring, and expand our understanding of potential mother–offspring conflict.

## 3 ERROR MANAGEMENT THEORY AND A COST–BENEFIT PERSPECTIVE OF VERTEBRATE MATERNAL STRESS

Error management theory, an evolutionary perspective based on signal detection theory (Box 1), provides a formal theoretical framework for evaluating how organisms (including humans) should make decisions amidst uncertainty (Haselton & Buss, 2000; Johnson, Blumstein, Fowler, & Haselton, 2013; Swets, 1992). EMT has been successfully used to examine many biological phenomena, such as plant defense mechanisms against herbivores (Orrock et al., 2015), mate‐selection behavior (Haselton & Buss, 2000), deception in animal communication (Wiley, 1994), optimal antipredator behavior (Bouskila & Blumstein, 1992), and defense mechanisms in human health and disease (Nesse, 2005). The broad applicability of EMT is possible because it comprises the basic components common to most decisions made by microbes, plants, and animals: based on some amount of information regarding the likelihood of an event, an organism chooses to respond (or not to respond), and that response (or lack of response) has some probability of being incorrect in two distinct ways (analogous to type I and type II errors in standard hypothesis testing). Importantly, EMT posits that, when the two different types of error have different fitness costs (or benefits), selection will favor individuals that err toward making the least costly error to avoid making a costlier one.

Since the quality of the maternal environment can often be indicative of the conditions experienced by her offspring, EMT may be a particularly tractable framework for considering the adaptive significance of maternal‐stress effects given the framework's ability to compare the relative fitness costs and benefits of phenotypic changes within relevant future environments. Specifically, EMT can be used to assess whether the effects of maternal glucocorticoids on offspring phenotype generate relatively better (benefits) or worse (costs) fitness outcomes for mothers or offspring depending on the relative match of that adjusted phenotype to the future environment (Figure 1). Because future conditions cannot be predicted with complete accuracy, maternal‐stress effects can be incorrect in two ways. First, exposure to elevated maternal stress may induce a phenotypic response in offspring but the future environment that they encounter is not stressful, a false‐positive error expected to reduce offspring fitness compared to an unaltered offspring in that benign environment. Second, elevated maternal stress does not induce a phenotypic response in offspring and the future environment encountered by the offspring is stressful, a false‐negative error expected to reduce offspring fitness compared to an altered offspring in that stressful environment. Effects of maternal stress on offspring can in turn also be correct (i.e., with no associated error) in two distinct ways, collectively generating four possible offspring phenotype‐postnatal environment scenarios (Table 1, Figure 1): (1) unaltered offspring phenotype in a benign postnatal environment (no error), (2) altered offspring phenotype in a benign environment (error of unnecessary offspring modification), (3) unaltered offspring phenotype in a stressful environment (error of failing to modify offspring when necessary), and (4) altered offspring phenotype in a stressful environment (no error). Although EMT typically focuses on the costs and benefits of errors in affecting optimal decision making, within our framework, it is the costs and benefits of the actual decisions that are ultimately important and which influence the evolution of maternal‐stress effects (Box 1). Our framework is also cast in terms of offspring that may inhabit a future environment that is either benign or stressful. Although this dichotomous classification may suffice to capture relevant differences in many species (especially over the relatively short window early in life when environmental stressors are likely the biggest agents of offspring mortality), we note that the main conclusions of our work also apply in cases where offspring may inhabit environments that vary greatly in their stressfulness (Nesse, 2005). As such, our framework shows how the influence of maternal stress can be adaptive even when the stress‐induced phenotype of the offspring is not a perfect match to the environment (i.e., it demonstrates how seemingly maladaptive offspring phenotypes are actually adaptive when we incorporate the reality of an uncertain future and the likelihood of different error costs over time; Box 2).

Box 2 **Error management helps resolve the value of maternal stress**

1 Empirical evidence from ecological studies support the idea that maternal‐stress effects can be adaptive if the quality of the mother's environment predicts (i.e., matches) that of its offspring (i.e., a true negative or true positive), but maladaptive if it does not (i.e., false negative or false positive; Love & Williams, 2008; Sheriff & Love, 2013; Uller et al., 2013). The overall outcome is a dichotomous value of maternal stress. For example, snowshoe hares exhibit a 10‐year population cycle with their main predator, Canada lynx (Krebs et al., 1995). During the decline phase of their population cycle (when their population size is declining from its peak), hares experience extreme predation risk from lynx and exhibit increases in maternal glucocorticoids (Sheriff, Krebs, & Boonstra, 2011). These elevations in maternal glucocorticoids result in smaller, lighter offspring that have elevated hormonal responsiveness to a stressor, but which are assumed to be adapted to the high predation environments the offspring encounter (Sheriff, Krebs, & Boonstra, 2009; Sheriff et al., 2010). Although these modified offspring born during the decline phase encounter extreme predation risk from lynx, this is not the case for offspring that are born at the end of the decline phase or during the low phase of their population cycle (when population size is at its nadir; Sheriff et al., 2011). Thus, exposure to maternal stress may cause adaptive changes in offspring during the decline phase and yet seemingly maladaptive effects in offspring during the low phase because it seems to poorly match the environmental conditions the offspring will experience at independence (a low predation environment). However, when considered in our EMT‐based framework, the costs of the potential errors must be compared (i.e., the fitness value of a false positive vs. a false negative). Given this perspective, it is likely that maternal stress is adaptive throughout the hare cycle; living in a benign (low predation) environment as an altered offspring is likely far less costly than living in a predator‐rich environment as an unaltered offspring; that is, lower reproduction vs. quick death. In other words, the fitness costs of being an altered hare during the low phase when predation risk is low are likely outweighed by the fitness benefits of being an altered hare during the decline phase when predation risk is high. Thus, through the lens of EMT, the correct assessment of the relative adaptive function of maternal‐stress effects can be made since the EMT framework provides the relative fitness outcomes across various future environments.

**Table 1:** Fitness outcomes of maternal‐stress effects should be compared across all scenarios within a 2 × 2 framework, representing the four possible outcomes when offspring phenotype may (or may not) be modified in a way that does (or does not) match the future environment. For simplicity, we label the environment experienced by the mother or her offspring as “Stressful” (high levels of glucocorticoids relative to the species‐typical levels) or “Unstressful.” In general, we anticipate fitness rankings of *F*_TN_ > *F*_TP_ > *F*_FP_ > *F*_FN_ or *F*_TN_ > *F*_FP_ > *F*_TP_ > *F*_FN_, which of these is accurate depends upon the relative costs of false‐positive (FP) errors and true positive (TN) outcomes. Importantly, regardless of the relative fitness values of *F*_TP_ and *F*_FP_, we always expect *F*_FN_ to have the least fitness (and often by a substantial margin), such that error management would predict that mothers would produce offspring that are least likely to experience this error (i.e., mothers should err toward producing altered offspring to reduce the likelihood of failing to produce altered offspring that later experience a highly stressful environment). In general, we expect that many situations exist where offspring experience environments that are well‐approximated by a simple dichotomy of stressful vs. benign environments (especially over the relatively brief window early in life where offspring survival is typically most constrained). However, we note that the general predictions of the model still follow in cases where offspring may experience a range of stresses in the natal environment (so that the natal environment is not well described by a simple stressful/unstressful classification). As long as the fitness costs of the two types of error are asymmetrical and current information has some predictive utility for future conditions, we expect selection to favor maternal‐stress effects that lead to modified offspring when the costs of making unnecessarily altered offspring are much lower than the costs of failing to modify offspring then future stress is imminent (Nesse, 2005)

		Environment experienced by offspring	
		Unstressful	Stressful
Maternal‐stress alteration of offspring phenotype	Unstressful	Unaltered offspring in benign environment, no error *True Negative (TN)*	Error of failing to modify offspring when necessary *False Negative (FN)*
	Stressful	Error of unnecessary offspring alteration *False Positive (FP)*	Altered offspring in stressful environment, no error *True Positive (TP)*

## 4 PREDICTING THE RELATIVE STRENGTH OF VERTEBRATE MATERNAL‐STRESS EFFECTS

Our framework provides further predictive power enabling researchers to forecast variation in the influence of maternal stress on offspring phenotype across taxa and life histories (Box 3). First, EMT provides a means for predicting the threshold at which a developmental decision will be made within a given species (Box 1), where the decision is the phenotypic response of offspring (more akin to a mechanistic reaction than a typical decision) and the threshold is the level of maternal stress (i.e., glucocorticoid hormones) at which this response occurs in offspring. For example, our framework predicts that species that experience much greater costs to producing an unaltered offspring in the face of a stressful environment (i.e., a false‐negative error) should have a much lower maternal stress threshold at which offspring phenotypic response occurs compared to a species where the costs of false‐negative errors are lower (or the costs of false‐positive errors are higher). Highly vulnerable prey, such as species with type III survivorship curves (i.e., very low offspring survivorship), should respond at a much lower maternal‐stress threshold compared to prey species that are not as vulnerable to predation, such as those with type I or II survivorship curves (i.e., very high or moderately higher offspring survivorship, respectively). This relationship may also be influenced by where species fall along the precocial–altricial axis of life‐history variation (precocial and altricial offspring differ in the duration of postnatal parental care). We would expect species producing more precocial offspring (requiring shorter periods of postnatal care) to respond at a lower maternal‐stress threshold than species producing more altricial offspring (requiring longer periods of postnatal care). This is because the greater duration of parental care in the more altricial species may offer an opportunity to reduce the costs of a mismatch of offspring phenotype and postnatal environment (i.e., an error that can somewhat be corrected). For example, in both laboratory studies of rats and field studies of birds, maternal stress can alter offspring phenotype; however, postnatal maternal/parental care can reverse or enhance these effects or can modify an unmodified neonate's phenotype (Love & Williams, 2008; Meaney et al., 2007). All of which has the potential to reduce the costs of mismatch errors (i.e., false negative/positive errors) in species that exhibit high degrees of parental care (e.g., primates or passerine bird species).

Box 3 **Predictions for variation in offspring response to maternal stress across life histories**

1

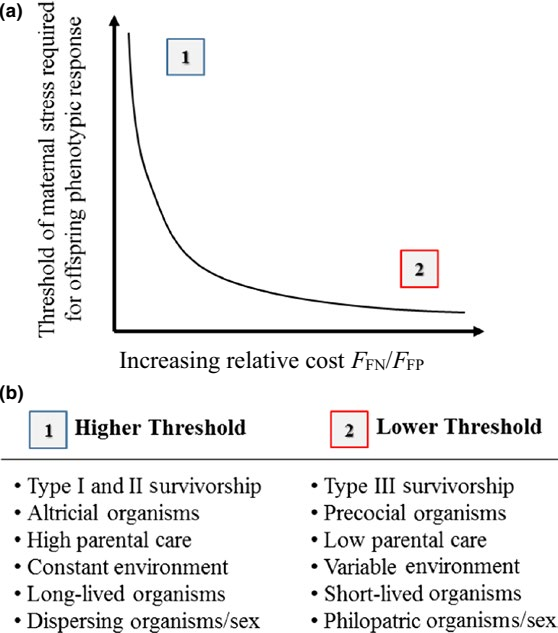

Error management theory can help inform qualitative predictions about the variation in the strength of influence of maternal stress on offspring phenotype (maternal‐stress effects) both among and within species and populations. (a) Focusing on situations where successful matches (i.e., true positive and true negative outcomes) have the same benefit, the relative cost of failing to modify offspring phenotype when necessary (false‐negative errors) compared to the cost of unnecessary modification in a benign environment (false‐positive errors) may drive the threshold at which an offspring's phenotype responds to maternal stress. (1) When costs of false‐negative errors are small relative to costs of false‐positive errors, we expect that offspring phenotype will only be modified at relatively high levels of maternal stress. Alternatively, (2) when costs of false‐negative errors are very large relative to costs of false‐positive errors (e.g., when highly lethal stressors are common in the offspring environment), we expect that offspring phenotype will be modified at relatively low levels of maternal stress. (b) We expect that particular life‐history traits, as well as particular ecological situations, will influence the amount (or threshold) of maternal stress required to initiate a change in offspring phenotype. (1) We expect relatively weak maternal‐stress effects for those organisms where there is (i) a low risk of offspring mortality (type I) or an equal risk of mortality across lifestages (type II), (ii) parental care to buffer offspring's exposure to the postnatal environment (altricial species), (iii) a relatively constant environment, and (iv) a significant disconnect between maternal and offspring environment (high‐dispersal or long‐lived species), (2) while we expect a lower threshold of response and relatively strong maternal‐stress effects in organisms which display opposing traits.

Our framework also predicts that maternal‐stress effects should be strongest in species where there is generally high spatial and/or temporal variation in stressors among generations but relative consistency in stressor magnitude and from the time of gestation through to offspring experience (early life); as these are situations where errors are most likely to occur over evolutionary time. As such, in species that experience periodic and/or unpredictable extremes in predator populations, food availability, or conspecific density among generations, but inhabit a relatively consistent environment from the time of gestation through to the early life of offspring (e.g., Dantzer et al., 2013; Kuijper, Johnstone, & Townley, 2014; Sheriff et al., 2010), we would expect a lower maternal‐stress threshold at which offspring phenotypic response occurs than in species with either high or low, but chronic, interannual exposure to such stressors. Examples of such species include snowshoe hares (*Lepus americanus*) or North American red squirrels (*Tamiasciurus hudsonicus*) in the Yukon, Canada, that can experience extreme interannual fluctuations in the abundance of predators, food, or conspecifics. These fluctuations in predation risk for snowshoe hares occur in a regular 10‐year cycle (Krebs et al., 1995) whereas the fluctuations in food and density in red squirrels (Boutin et al., 2006; Dantzer, Boutin, Humphries, & McAdam, 2012; Dantzer et al., 2013) are episodic, occurring every 3–4 years. For both species, the environments faced by offspring are qualitatively different (i.e., either benign or very stressful) and remain so for the course of offspring development (i.e., for the purposes of offspring survival, the environments remain either benign or stressful).

Our perspective may also provide insights into determining the origin of sex‐specific sensitivity to maternal or developmental stress (Box 3; Bale & Epperson, 2015; Brunton & Russell, 2010; Love et al., 2005). In species where there is disparity in the proximate or ultimate costs of raising a given sex, our framework predicts that the more expensive sex would have a lower threshold to respond to maternal stress given that the costs of errors would be higher compared to the less expensive sex (Love & Williams, 2008; Love et al., 2005). Likewise, in species with sex‐biased natal dispersal, our framework would predict that the dispersing sex should have a higher threshold to respond to maternal stress compared to the philopatric sex, given the reliability of the information about the future environment is lower in the dispersing sex (de Fraipont et al., 2000; Meylan & Clobert, 2005). This idea can be expanded to species with natal dispersal in general, and interestingly, to natal habitat preference induction, where dispersing individuals will select habitats that are most similar to their natal habitat (Davis & Stamps, 2004). This phenomenon would increase the match between the maternal and offspring environment and potentially reduce the cost of errors in offspring phenotype response.

## 5 MALADAPTIVE ERRORS IN RESPONSE TO NOVEL STRESSORS

As outlined above, species‐specific responses of offspring to maternal stress are likely to have been optimized by natural selection based on species life‐history and environmental variation experienced (Gluckman et al., 2005; Sheriff & Love, 2013). Thus, as with any adaptive phenotypic response that has been shaped by predictable variability in intrinsic or extrinsic environmental quality, there are potential negative implications with regard to human‐induced rapid environmental change (i.e., HIREC; Sih, 2013) many animals now face. Two likely scenarios have the potential to emerge as animals increasingly face novel stressors in their environments. First, these stressors will result in offspring phenotypes that may be maladapted to the novel stressor due to the presence of false‐positive errors. This circumstance is analogous to a situation where cues that once induced adaptive phenotypic plasticity now become unreliable (Trimmer, Ehlman, & Sih, 2017). For example, consider animals such as common lizards (*Zootoca vivipara*) in which maternal stress increases offspring propensity to disperse as an adaptive response to increasing predation risk (Bestion et al., 2015; Meylan & Clobert, 2005). If such animals now face a novel anthropogenic stimulus (e.g., traffic noise) that also induces maternal stress, the resultant offspring phenotype may exhibit a false‐positive error (since the stressor was not predation risk), and the cost of this error may now decrease (rather than increase) offspring fitness. Second, animals may not respond to a novel stressor if mothers do not perceive it as stressful (i.e., a false‐negative error). For example, mothers may be faced with novel introduced predators, but fail to perceive them as threatening (Sih et al., 2010), resulting in unaltered offspring phenotypes and likely lowered fitness in the new high predation environment. EMT predicts that animals will likely make maladaptive errors, in both direction and relative strength, to novel stressors since their decision bias (in our case maternal‐stress effects) was shaped over evolutionary time. This bias could then result in evolutionary traps (Schlaepfer, Runge, & Sherman, 2002) given present‐day environmental changes that may increase the degree of mismatch between the maternal and offspring environments or decrease the reliability of cues that mothers generate that offspring in turn may use to forecast the environments they will encounter at independence.

## 6 FUTURE DIRECTIONS: EXTENDING MODEL PREDICTIONS AND APPLICATIONS TO OTHER SYSTEMS

While we focus on maternal‐stress effects in vertebrates, maternal effects via other mechanisms have been documented in a variety of systems, including plants (e.g., Schuler & Orrock, 2012) and arthropods (Mousseau & Dingle, 1991) as well as reptiles, amphibians, birds, and mammals (Mousseau & Fox, 1998; Uller, 2008). Several of the key predictions from our framework may extend to these groups as well, where they can be useful in generating both species‐specific predictions and testing environmentally specific hypotheses in the field. For example, it is well established that plants exhibit a multitude of transgenerational effects in response to a diverse array of environmental stressors, including herbivory, temperature, and resource‐related stress (Agrawal, 2001; Crisp et al., 2016; Walter, Harter, Beierkuhnlein, & Jentsch, 2016). EMT could be used to broadly examine the environmental and life‐history conditions under which these transgenerational effects are adaptive. More specifically, EMT would predict that for plants that produce small seeds (e.g., often annual plants), transgenerational maternal‐stress effects might be triggered at relatively modest levels of environmental stress, since the costs of false‐negative errors may be very high for small‐seeded species whose seedlings do not have large energy or resource reserves for tolerating stress. On the other hand, plant species producing larger seeds should pay lower costs for false‐negative errors (because seedlings have greater reserves to help ameliorate the cost of a false‐negative error), and EMT would predict a reduced response of seed phenotype to maternal stress.

In many plant species, as well as aquatic or terrestrial invertebrates and vertebrate species, that produce numerous, low‐cost propagules in their lifetime, offspring may experience very high mortality during development. As such, these species may adopt a bet‐hedging, rather than preparative, strategy with regard to future stressors (Herman, Spencer, Donohue, & Sultan, 2014), where current stress signals are ignored even if they are predictive of future stress. An important future direction (Box 4) will be examining predictions generated with EMT in these species.

Box 4 **Outstanding questions in integrating EMT into maternal‐stress effects**

1

1. Are the fitness benefits of maternal stress dependent upon the environment offspring experience at independence? It is important to quantify effects of stress‐induced phenotypes in offspring in both stressful and nonstressful environments to fully characterize the costs and benefits of offspring phenotypes modified by maternal stress.
2. Are the effects of maternal stress on offspring characteristics dependent upon the ecological trigger inducing maternal stress? Environmental stressors such as reduced food availability or high predation risk can both increase maternal glucocorticoids, but it is unclear whether the effects of elevated maternal glucocorticoids on offspring phenotype are the same for these different ecological triggers of maternal stress.
3. Do offspring or mothers control the point at which elevated maternal glucocorticoids alter offspring traits? Offspring and mothers can be in conflict with how maternal stress alters offspring traits, can offspring resist the effects of maternal glucocorticoids and, if so, how?
4. What role do fathers play in this EMT view of maternal‐stress effects? In species with biparental care, fathers could buffer the effects of maternal stress on offspring by modifying the cost of false‐negative or false‐positive errors. Fathers may also buffer the environment experienced by the mother, reducing her level of stress.
5. How does anthropogenic environmental change modify the occurrence of false negatives and false positives relative to environments over a species’ evolutionary past? For example, the mismatch between maternal and offspring environments is likely elevated due to human‐induced rapid environmental change, which should increase the frequency of errors. Moreover, different kinds of human‐induced rapid environmental change (i.e., HIREC, see Sih, 2013) could generate mismatches that vary in type and magnitude. For instance, introduced predators may increase false‐negative errors because they are not recognized as dangerous and do not cause maternal stress. Resource subsidies from ephemeral anthropogenic habitats (e.g., agricultural fields) might lead to increased false‐positive errors because food is plentiful for mothers, but may not be for their offspring.
6. How effectively does the EMT framework capture transgenerational maternal‐stress effects for organisms (e.g., many plants, invertebrates, and vertebrates) that produce very large numbers of propagules/offspring? Are transgenerational EMT effects, which would lead to directional shifts in offspring phenotype (i.e., deterministic maternal effects, sensu Proulx & Teotónio 2017) more commonly observed for such species than strategies based upon randomly increasing the range of phenotypes exhibited by offspring (diversifying bet‐hedging via random maternal effects; Proulx & Teotónio, 2017)?
7. If mothers bear substantial costs for unnecessary modifications of offspring phenotype (false positives), how does this alter the predictions of our EMT framework? We focus on offspring fitness, but mothers may suffer substantial fitness costs for true or false positives and this could affect the predicted fitness rankings of each scenario shown in Table 1.

Overall, we have chosen to outline the EMT framework focused primarily on vertebrate taxa that experience fluctuating environments in which we expect parental/maternal effects to have a large influence on offspring phenotype relative to other sources of variation (Leimar & McNamara, 2015) and compared to other mechanisms of dealing with fluctuating environments such as bet‐hedging (Proulx & Teotónio, 2017). Although we have focused on maternal‐stress effects in vertebrates, we expect that future studies in any organism could use the same framework, substituting their own taxa‐ or species‐specific mechanism or signal of environmental quality that a parent can pass to their offspring. Studies expanding this framework to other organisms are both greatly needed and have the power to more robustly test EMT within this maternal‐effect framework.

## 7 CONCLUDING REMARKS

When viewed from an EMT perspective, the adaptive nature of seemingly maladaptive maternal stress effects becomes more readily apparent (Box 1). The EMT framework outlined here provides a means to reconcile the persistence of the sometimes seemingly maladaptive role of maternal stress (Box 2), an array of hypotheses (Box 3), and generates additional functional questions (Box 4) to help us further characterize and appreciate the tremendous variation in phenotypes and fitness outcomes that are often observed. It further allows us to better predict how animals may (or may not) respond to novel stressors. An important pragmatic benefit of our EMT approach is that, unlike some theoretical models, it can provide qualitative predictions that can be readily tested by experimental manipulation of components known to alter vertebrate maternal stress and quantifying how this alters offspring phenotype, and the relative performance and fitness outcomes. We expect that new studies adopting experimental manipulations of maternal stress across related species that exhibit a diversity of life histories and across a continuum of environmental fluctuations will be particularly useful in testing the predictions of EMT to explain the adaptive role of maternal stress. Expanding the EMT framework to other taxa is especially needed to test both the generality and the robustness of EMT for predicting transgenerational maternal‐stress effects in a variety of ecological and life‐history contexts.

## CONFLICT OF INTEREST

None declared.

## AUTHOR CONTRIBUTIONS

MJS formulated the original concept and led the writing of the manuscript; JLO, BD, and OPL provided significant intellectual contributions, drafted portions of text and figures, and contributed to manuscript revision.
